# Immunohistochemical Ki67 after short-term hormone therapy identifies low-risk breast cancers as reliably as genomic markers

**DOI:** 10.18632/oncotarget.15385

**Published:** 2017-02-16

**Authors:** Takayuki Iwamoto, Toyomasa Katagiri, Naoki Niikura, Yuichiro Miyoshi, Mariko Kochi, Tomohiro Nogami, Tadahiko Shien, Takayuki Motoki, Naruto Taira, Masako Omori, Yutaka Tokuda, Toshiyoshi Fujiwara, Hiroyoshi Doihara, Balazs Gyorffy, Junji Matsuoka

**Affiliations:** ^1^ Department of Breast and Endocrine Surgery, Okayama University Hospital, Okayama, Japan; ^2^ Department of Gastroenterological Surgery, Okayama University Hospital, Okayama, Japan; ^3^ Division of Genome Medicine, Institute for Genome Research, Tokushima University, Tokushima, Japan; ^4^ Department of Breast and Endocrine Surgery, Tokai University School of Medicine, Kanagawa, Japan; ^5^ Department of Pathology, Okayama University Hospital, Okayama, Japan; ^6^ MTA TTK Lendület Cancer Biomarker Research Group, Budapest, Hungary; ^7^ 2nd Department of Pediatrics, Semmelweis University, Budapest, Hungary; ^8^ Department of Palliative and Supportive Medicine, Okayama University Hospital, Okayama, Japan

**Keywords:** short-term hormone therapy, IHC Ki67, genomic marker, breast cancer

## Abstract

**Background:**

The purpose of this study was to test whether immunohistochemical (IHC) Ki67 levels after short-term preoperative hormone therapy (post-Ki67) predict similar numbers of patients with favorable prognoses as genomic markers.

**Results:**

Thirty paired cases (60 samples) were enrolled in this study. Post-Ki67 levels were significantly lower than pre-treatment Ki67 levels (*P* < 0.001). Post-Ki67 predicted more low-risk cases (83.3%, 25/30) than pre-genomic surrogate signature(GSS) (66.7%: 20/30), but the difference in predictive power was not significant (*P =* 0.233). Proliferation (*MKI67*, *STK15*, *Survivin*, *CCNB1*, and *MYBL2*) and estrogen (*ER*, *PGR*, *BCL2*, and *SCUBE2*) related signatures were significantly downregulated after therapy (*P* < 0.001 and 0.041, respectively).

**Materials and Methods:**

Core needle biopsy specimens of primary breast cancer were collected at Okayama University Hospital from hormone receptor-positive and human epidermal growth factor 2-negative patients that subsequently received two weeks of neoadjuvant hormone therapy. Paired post-treatment specimens from surgical samples were also collected. IHC Ki67 levels and GSS were compared between pre- and post-hormone treatment samples. Changes of gene expression pattern in short-term hormone therapy were also assessed.

**Conclusions:**

IHC based post-Ki67 levels may have distinct predictive power compared with the naïve IHC Ki67. Future studies with larger cohorts and longer follow-up periods may be needed to validate our results.

## INTRODUCTION

Genomic signatures can predict prognoses of cancer patients to indicate which patients do not need chemotherapy and which are most likely to respond to chemotherapy. These signatures are more precise than classical clinicopathological biomarkers, such as histological grade and immunohistochemical (IHC) assessment of Ki67 among patients with hormone receptor (HR) positive (+)/human epidermal growth factor receptor 2 (HER2) negative (−) breast cancer [1]. The National Comprehensive Cancer Network (NCCN) guidelines recommend using a validated 21-gene reverse transcription polymerase chain reaction (RT-PCR) prognostic assay to guide treatment management when combining chemotherapy with standard hormone therapy [2]. Reducing unnecessary chemotherapy for HR+/HER2− breast cancer cases is one of the greatest advantages of using genomic signatures to design treatment practices [3]. However, the high cost of genomic signature testing (*e.g*. the Oncotype DX^®^ test costs currently $4,175) limits clinical access to this diagnostic technology in many countries outside of the US and Western Europe.

Dowsett et al recently showed that an inexpensive biomarker assay using IHC Ki67 after short-term preoperative hormone therapy had improved predictive power for breast cancer prognosis [4]. IHC Ki67 levels after short-term hormone therapy were significantly lower than naïve IHC Ki67 levels [5]. However, it is unknown if IHC Ki67 after short-term hormone therapy (post-Ki67) can predict favorable prognoses among HR+ breast cancer cases as accurately as genomic signatures can. No clinical studies have examined both genomic signatures and post-Ki67 levels to allow the predictive functions of the distinct markers to be directly assessed on the same cohort of patients.

The purpose of this study was to compare the predictive power of post-Ki67 to known genomic markers and to assess changes of gene expression pattern associated with short-term hormone therapy.

## RESULTS

Thirty paired cases (60 samples) with HR+/HER2− breast cancer were enrolled in this study. Patient characteristics are shown in Table 1. Of these, 14 cases (46.7%) were clinical T1 breast cancers and no patients had clinical lymph node metastasis. All cases were HR+ and HER2−, and 25 cases (83.3%) were PgR+. Over half of the cases (53.3%) were histological grade I. Eleven cases (36.7%) received tamoxifen and the remaining 19 cases received letrozole as the preoperative short-term hormone therapy.

**Table 1:** Patient characteristics*

Age	Average (min.-max)	59.5 (40–88)
	Number of samples	%
cTumor size		
T1	14	46.7%
T2	15	50.0%
T3	1	3.3%
T4	0	0.0%
cN		
Positive	0	0.0%
Negative	30	100.0%
ER		
Positive	30	100.0%
Negative	0	0.0%
PgR		
Positive	25	83.3%
Negative	5	16.7%
HER2		
Positive	0	0.0%
Negative	30	100.0%
Historogical grade		
I	16	53.3%
II	11	36.7%
III	2	6.7%
Unknown	1	3.3%
Hormone therapy		
Tamoxifen	11	36.7%
Letrozole	19	63.3%
Operation		
Total mastectomy	12	40.0%
Partial mastectomy	18	60.0%

Abbreviations: *c: Clinical; ER: Estrogen receptor; PgR: progesterone receptor; HER2: human epidermal growth factor receptor 2.

### IHC Ki67 and genomic surrogate signature (GSS) after short-term hormone therapy

**Figure 1:**
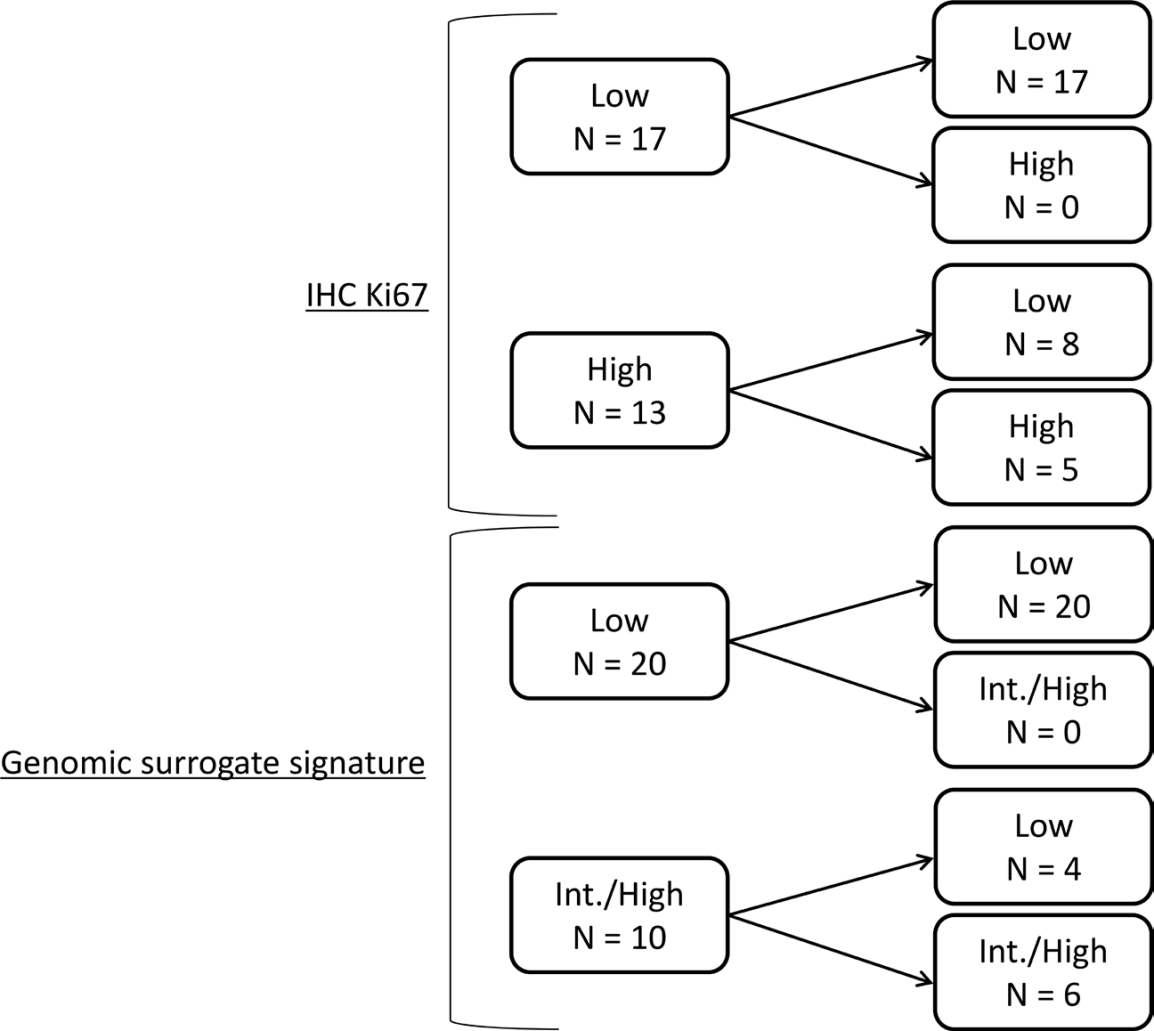
Differences in risk categories by pre- and post-treatment markers

**Figure 2:**
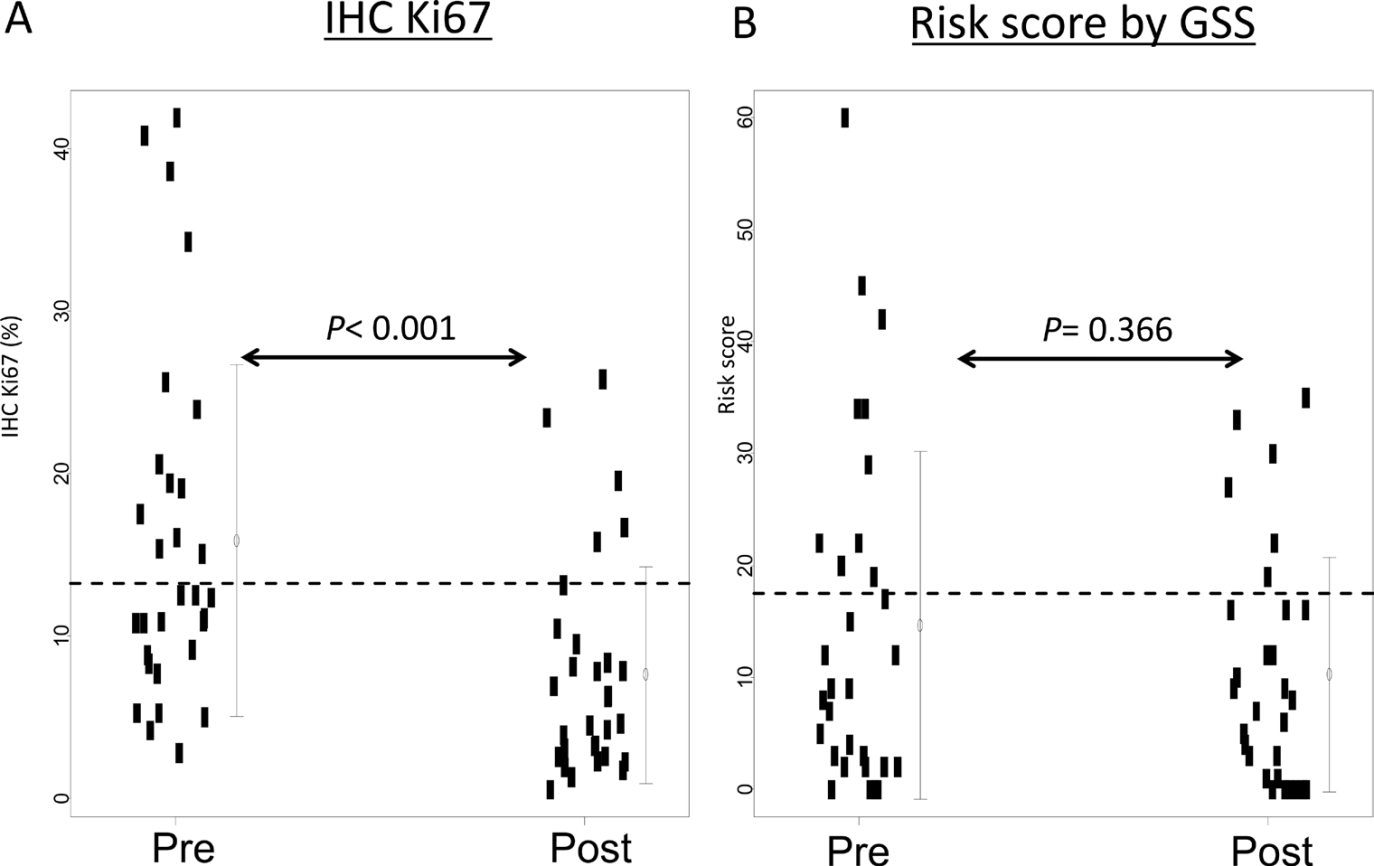
IHC Ki67 levels and web-based risk scores before and after short-term hormone therapy (**A**) The IHC Ki67 level was defined as the percentage of Ki67-positive cells. The dotted line shows the cutoff IHC Ki67 level at 13.25%. (**B**) The web-based risk score was simulated [17]. The dotted line shows the cutoff for low and intermediate risk groups. *P* values were calculated by the Wilcoxon test.

Seventeen cases (56.6%) were assigned to the low-risk group according to pre-treatment Ki67 (pre-Ki67) levels and 25 cases (83.3%) were assigned to the low-risk group according to post-Ki67 levels (Figure 1). The average pre- and post-Ki67 levels were 15.9% and 7.6%, respectively. Pre-Ki67 levels were significantly higher than post-Ki67 (*P* < 0.001) (Figure 2A). Pre-treatment GSS stratified 20 cases (66.7%) into the low-risk group. Twenty-four cases (80%) were classified as low-risk by post-treatment GSS. Pre- and post-therapy recurrence scores according to GSS were not significantly different (*P* = 0.366) (Figure 2B). Post-Ki67 predicted more low-risk cases (83.3%, 25/30) than pre-GSS (66.7%: 20/30), but the difference in predictive power was not significant (*P* = 0.233). All cases assigned to the low-risk group by pre-Ki67 (*n* = 17) and pre-GSS (*n* = 20) were again stratified into low-risk group by post-treatment Ki67 and GSS. Conversely, more than half of cases assigned to the high-risk group by pre-Ki67 (8/13) and 40% (4/10) of cases assigned to the intermediate- or high-risk groups by pre-GSS were reclassified into low-risk group by post-treatment markers.

### Changes of gene expression pattern associated with short-term hormone therapy

We compared gene expression levels of four clinically important genes before and after hormone therapy. After short-term hormone therapy, *PGR* (208305_at) and *MKI67* (212020_s_at) were significantly downregulated (*P* = 0.043 and *P* < 0.001, respectively), and *ESR1* (205225_at) levels were marginally lower (*P* = 0.055) (Supplementary Figure 1). *ERBB2* (216836_s_at) levels were unaffected by the hormone treatment (*P* = 0.458).

**Figure 3:**
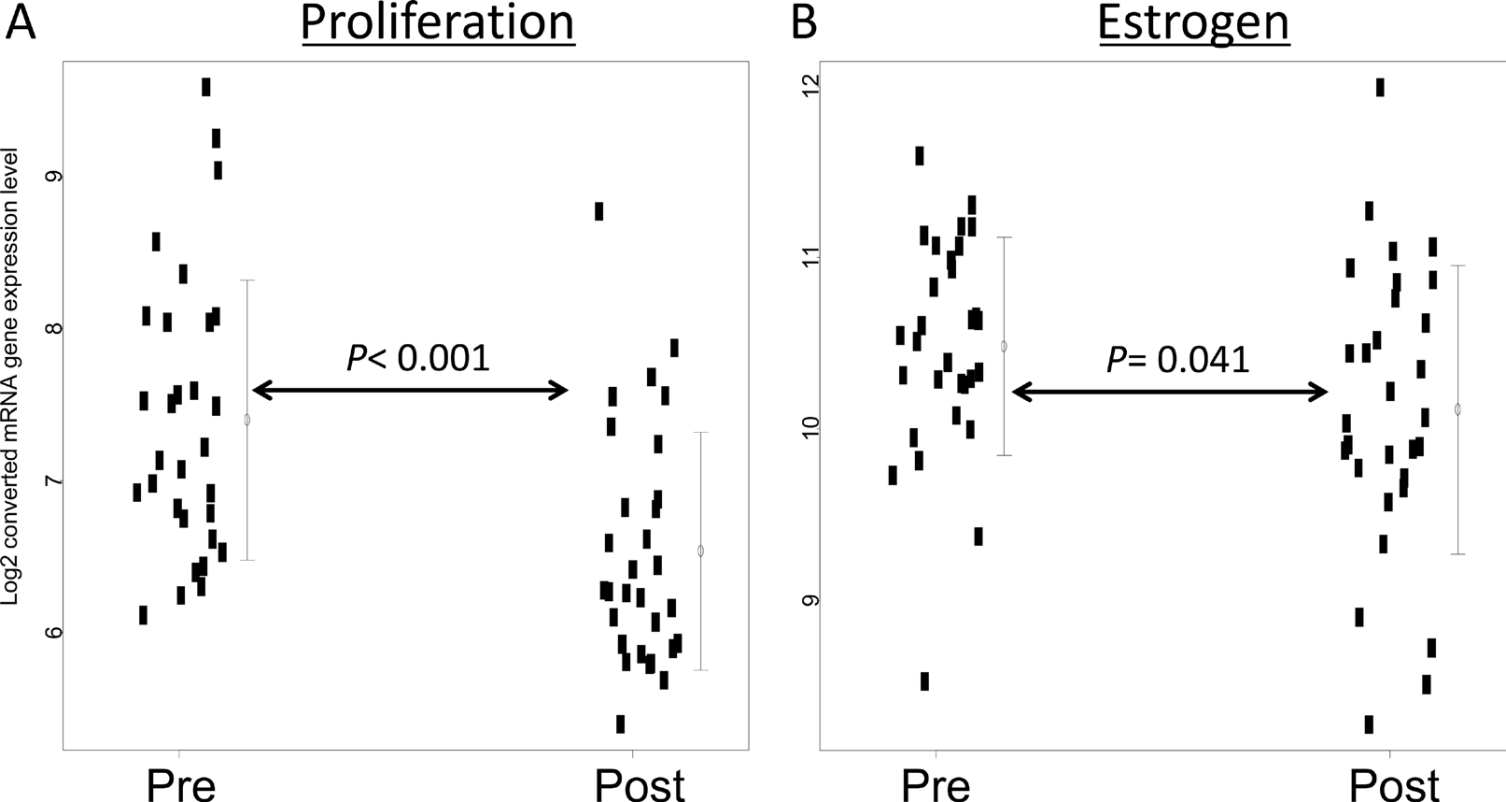
Gene expression before and after short-term hormone therapy (**A**) Proliferation-associated (*MKI67, STK15, Survivin, CCNB1*, and *MYBL2*) and (**B**) estrogen-associated (*ER, PGR, BCL2*, and *SCUBE2*) genes refer to average expression values [12]. *P* values were calculated by the Wilcoxon test.

Next, we assessed four biological functions and three independent genes from the 21-gene assay before and after therapy. Proliferation (*MKI67*, *STK15*, *Survivin*, *CCNB1*, and *MYBL2*) and estrogen (*ER*, *PGR*, *BCL2*, and *SCUBE2*) were significantly downregulated after short-term hormone therapy (*P* < 0.001 and 0.041, respectively) (Figure 3). Two biological functions (invasion and HER2) and three independent genes (*GSTM1*, *CD68*, and *BAG1*) were unaffected by the treatment (Supplementary Figure 2).

## DISCUSSION

Short-term hormone therapy is easily accessible, affordable, and has minimal side effects. Indeed, all patients in this study completed two weeks of hormone therapy without any side effects. Our study has notable findings regarding the impact of preoperative short-term hormone therapy for patients with HR+/HER2− breast cancers.

First, post-Ki67 levels classified as many low-risk cases as genomic markers. This finding suggests that post-Ki67 can be used as a reliable substitute for genomic testing. While genomic testing is likely to improve survival and quality-adjusted life expectancy in patients with high recurrence risks [6, 7], the cost of genomic testing limits its use outside the US and parts of Europe. Thus, identifying a low-cost marker such as IHC Ki67 that is as reliable as traditional genomic testing is critical to improve global cancer treatment strategies. Because no risk assessment method provides perfect predictions, additional biomarkers for HR+/HER2− breast cancers can help determine when patients classified as high-risk do not need chemotherapy or when patients classified as low-risk do require chemotherapy. In this study, short-term preoperative hormone therapy stratified all low-risk patients into the low-risk group according to pre-treatment biomarker levels. However, some patients designated as high-risk by pre-treatment biomarkers were classified to the low-risk group by post-treatment biomarkers. This finding suggests that our strategy might be more useful in determining when high-risk patients do not require chemotherapy than in revealing low-risk patients who do need chemotherapy. In this study we merged intermediate with the high risk group in the GSS. From the previous paper for the prospective study “TAILORx”, cases with a recurrence score of 0 to 10 with endocrine therapy alone without chemotherapy should have a very low risk of recurrence, indicating they do not need adjuvant chemotherapy [8], although we have no prospective data to skip chemotherapy for cases with intermediate risk at present. We need the additional evidence whether cases with intermediate risk can skip chemotherapy or not. This coming evidence may effect on our results.

Second, preoperative hormone therapy for only two weeks stimulated dynamic changes in gene expression pattern. We observed proliferation and estrogen associated genes were significantly downregulated after short-term hormone therapy. Consistent with our findings, Dowsett et al report that proliferation-associated genes and classical estrogen-dependent genes were strongly downregulated, and that higher expression of immune-related genes was associated with poorer responses [9]. Miller et al also found dynamic patterns of differential expression of proliferation and ER genes [10]. Two weeks of preoperative hormone therapy is trivial compared to the traditional five years of postoperative hormone treatment. However, there may be biological differences between adjuvant therapies that target unknown cancer stem cells and neoadjuvant therapies that target existing tumors. The results of the ongoing POETIC trials to determine whether preoperative endocrine therapy improves breast cancer treatment outcomes are eagerly awaited [11].

Interestingly, the genomic changes observed after hormone therapy do not align perfectly with a previous study [12]. This disagreement may have arisen because the first-generation genomic marker that was found to be highly associated with proliferation was composed of several functions and genes, and the previous study calculated risk scores as coefficients of those genes. We found that both proliferation and estrogen were significantly decreased after hormone treatments. While proliferation was positively correlated with the recurrence score, estrogen was negatively correlated with recurrence score [12]. In light of these conflicting findings, recurrence scores calculated by GSS might be relatively unaffected by short-term hormone therapy. These results are consistent with the previous paper [13]. The study by Ueno et al. compared the 21-gene assay Recurrence Score^®^(RS) between pre and post 6 months-hormone therapy and demonstrated no statistically significant difference in the hormone therapy (*P* = 0.484) [13].

A strength of this study is that both IHC Ki67 levels and genomic profiling data were available on paired samples. Therefore, the predictive power of IHC Ki67 and the genomic marker could be directly compared. However, this study has several limitations. The sample size of this study is small, and thus, our findings must be interpreted cautiously. Variability in IHC Ki67 counts between technical replicates and different pathologists were assessed, as reproducibility and consistency of IHC Ki67 testing are always debated [14, 15]. Heterogeneous treatments (tamoxifen or letrozole) may also affect the results. Furthermore, the tumor samples collected by core needle biopsy at diagnosis and during surgery could vary because of heterogeneity within the tumor. Different tissue samples from the same tumor may have different IHC levels and distinct gene expression profiles. Nevertheless, we believe that our findings are generalizable and are consistent with Ki67 levels and biological changes after short-term hormone therapy observed in other studies [4, 9].

In conclusion, our study assessed the effect of preoperative short-term hormone therapy on treatment outcomes and found that IHC based post-Ki67 levels may have distinct predictive power compared with the naïve IHC Ki67. Future studies with larger cohorts and longer follow-up periods may be needed to further test the efficacy of IHC Ki67 after short-term preoperative hormone therapy.

## MATERIALS AND METHODS

### Patient cohort and gene expression data

Core needle biopsy (Cx) with 14G or 16G for primary cancer specimens were collected at Okayama University Hospital from HR+/HER2− patients that subsequently received two weeks of neoadjuvant hormone therapy. Thirty clinical TNM stage I and II women were enrolled in this study. The study was approved by the Institutional Review Board and all patients signed informed consent forms. Patients received preoperative hormone therapy daily for two weeks before surgery. Premenopausal patients received tamoxifen (20 mg) and postmenopausal patients received letrozole (2.5 mg). All patients underwent a mastectomy or breast-conserving surgery. Surgical samples after treatment were also collected. Hormone and HER2 receptor statuses were determined in the diagnostic Cx specimens before hormone therapy. Cases with ≥1% positive nuclear staining for estrogen receptors (ER) or progesterone receptors (PgR) with IHC were considered hormone receptor-positive. Cases with either 0 or 1 positive IHC staining for HER2 or with a HER2 gene copy number < 2.0 by fluorescent *in situ* hybridization (FISH) analysis were considered HER2−. IHC staining for Ki67 established a proliferation index and was assessed pre- and post-neoadjuvant hormone therapy. The IHC Ki67 assay was performed using the MIB-1 antibody (Dako; Glostrup, Denmark) on the immunostainer system (Dako). Photomicrographs were taken under 40× magnification and the percentage of Ki67-positive cells was scored. Where possible, 1,000 malignant cells (at least 500 cells) were viewed. Replicate counts by two trained physicians were averaged to calculate % Ki67-positive cells. HR+/HER2− cases were divided into low and high Ki67 groups using the 13.25% Ki67 cutoff point as previously described by Cheang *et al*. [16].

Matching pre and post hormone therapy frozen specimens for gene expression analysis were collected into RNA and later stored at −80°C. RNA from breast specimens was isolated, and quantity and quality of the each RNA was using an Agilent 2100 Bioanalyzer (Agilent Technologies). Genome-wide expression levels of transcripts were analyzed using the Affymetrix U133A gene chips (Affymetrix) according to the manufacture's instructions. Complete gene expression data are available in the Gene Expression Omnibus (GEO) under accession number GSE80077.

### Bioinformatics and statistical analysis

We developed a genomic surrogate signature (GSS) simulated from Recurrence Online (http://www.recurrenceonline.com/) to calculate the recurrence score (0–100) and recurrence risk (low, intermediate, and high) using predefined sets of genes that were quantified by a cDNA microarray [17]. Breast cancers were stratified as low (score 0–17), intermediate (score 18–30), or high (score 31–100) risk using the GSS. First, we assessed how many patients were stratified to two groups (high or low risk group) by pre- or post-treatment GSS and IHC Ki67. We compared the recurrence score and IHC Ki67 levels before and after hormone therapy using a Wilcoxon test.

Next, we compared pre- and post-hormone therapy expression levels of four genes (*ER*, *PgR*, *HER2*, and *Ki67*) that are used clinically to inform breast cancer treatment strategies. Next, we measured expression changes of genes involved in key biological markers and functions using the 21-gene assay to investigate the effect of short-term hormone therapy on these genomic markers. The 21-gene assay was composed genes involved in four biological functions (Proliferation: *MKI67, STK15, Survivin, CCNB1*, and *MYBL2;* Invasion: *MMP11* and *CTSL2;* HER2: *GRB7* and *HER2;* Estrogen: *ER, PGR, BCL2*, and *SCUBE2*), three independent genes (*GSTM1, CD68*, and *BAG1*) and five reference genes [12]. We compared the average pre- and post-hormone therapy gene expression levels of each biological function and of the four genes.

All statistical analyses were performed using BRB-ArrayTools version 3.9.0a (http://linus.nci.nih.gov/BRB-ArrayTools.html) and R software version 2.7.2 (http://www.r-project.org). Two-sided *P* values ≤ 0.05 were considered statistically significant.

## SUPPLEMENTARY MATERIALS FIGURES


