# Supplementary material for: Immunohistochemical Ki67 after short-term hormone therapy identifies low-risk breast cancers as reliably as genomic markers
Source: Oncotarget. 2017 Feb 16;8(16):26122–8. doi: 10.18632/oncotarget.15385 (PMC5432244; doi:10.18632/oncotarget.15385)
Supplement: Supplementary file 1 [file oncotarget-08-26122-s001.pdf]

Immunohistochemical Ki67 after short-term hormone therapy identifies low-risk breast cancers as reliably as genomic markers

Supplementary Materials

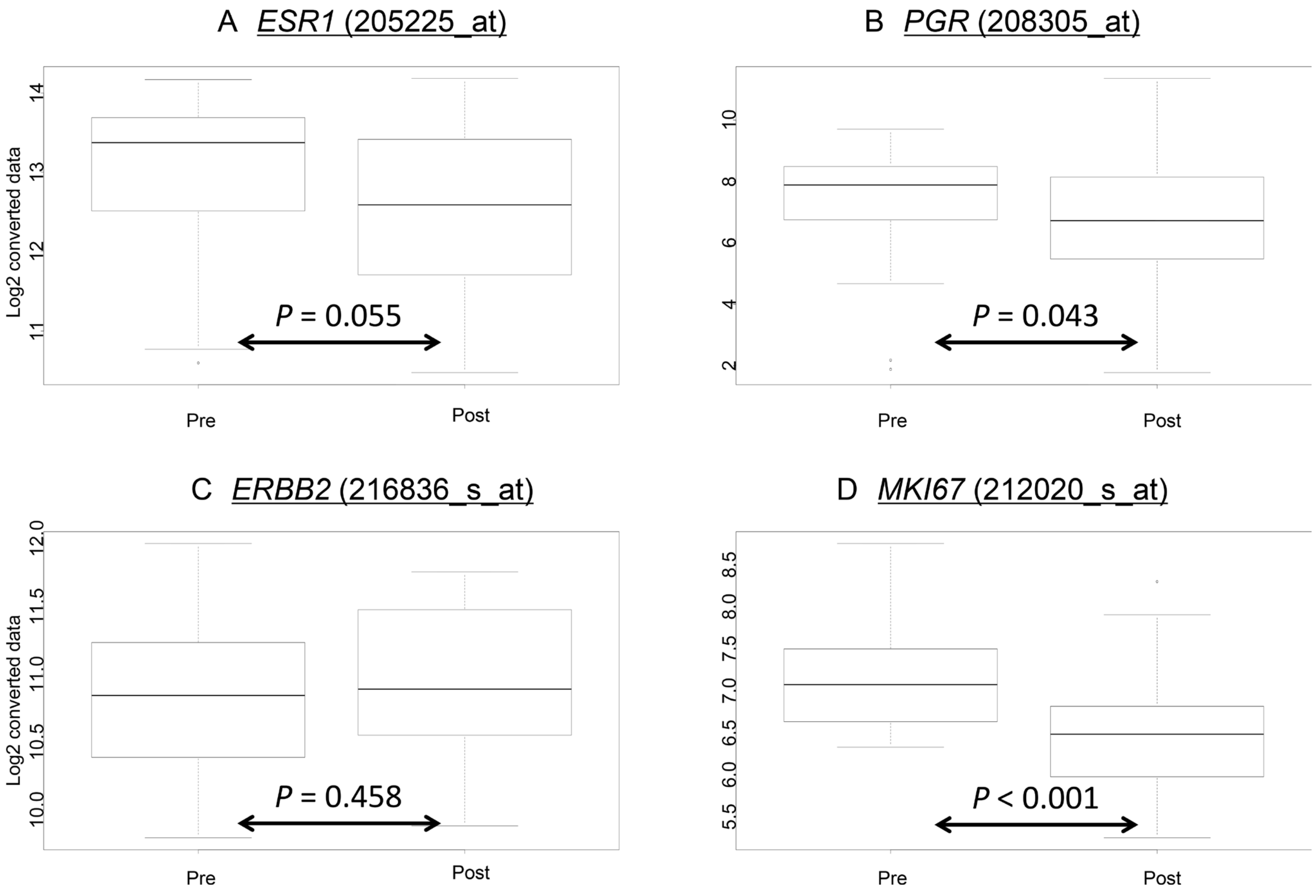

Supplementary Figure 1: Gene expression before and after short-term hormone therapy. *P* values were calculated by the Wilcoxon test.

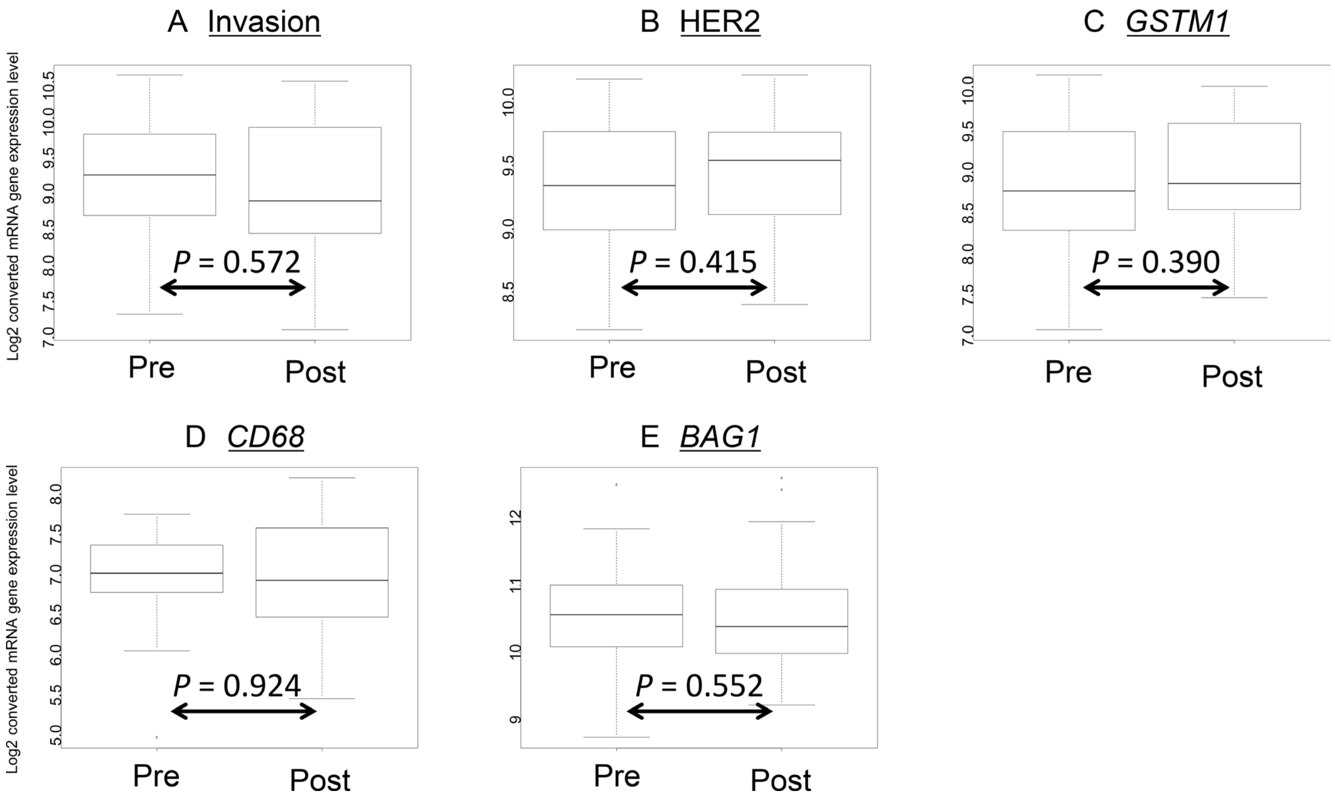

Supplementary Figure 2: Gene expression before and after short-term hormone therapy. (**A**) Invasion-associated (*MMP11* and *CTSL2*) and (**B**) HER2-associated (*GRB7* and *HER2*) genes refer to average expression values [12]. *P* values were calculated by the Wilcoxon test.
